# CS-KG 2.0: A Large-scale Knowledge Graph of Computer Science

**DOI:** 10.1038/s41597-025-05200-8

**Published:** 2025-06-09

**Authors:** Danilo Dessí, Francesco Osborne, Davide Buscaldi, Diego Reforgiato Recupero, Enrico Motta

**Affiliations:** 1 Department of Computer Science, College of Computing and Informatics, University of Sharjah, Sharjah, UAE; 2 The Open University, Knowledge Media Institute, Milton Keynes, UK; 3 Milano Bicocca University, Department of Business and Law, Milan, Italy; 4 Sorbonne Paris Nord University, Laboratoire d’Informatique de Paris Nord, Paris, France; 5 University of Cagliari, Mathematics and Computer Science Department, Cagliari, Italy

**Keywords:** Research data, Technology

## Abstract

The rapid evolution of AI and the increased accessibility of scientific articles through open access marks a pivotal moment in research. AI-driven tools are reshaping how scientists explore, interpret, and contribute to the body of scientific knowledge, offering unprecedented opportunities. Nonetheless, a significant challenge remains: dealing with the overwhelming number of papers published every year. A promising solution is the use of knowledge graphs, which provide structured, interconnected, and formalized frameworks that improve the capabilities of AI systems to integrate information from the literature. This paper presents the last version of the Computer Science Knowledge Graph (CS-KG 2.0), an extensive knowledge base generated from 15 million research papers. CS-KG 2.0 describes 25 million entities linked by 67 million relationships, offering a nuanced representation of the scientific knowledge within the field of computer science. This innovative resource facilitates new research opportunities in key areas such as analysis and forecasting of research trends, hypothesis generation, smart literature search, automatic production of literature review, and scientific question-answering.

## Background & Summary

We are at a crucial juncture in history, characterized by the rise of groundbreaking artificial intelligence tools for text analysis and the increased accessibility of scientific articles through open access. This progress has the potential to radically transform scientific research and how scientists engage with and contribute to scientific literature. The Artificial Intelligence (AI) community is actively working on sophisticated methods for smart and tailored literature searches^1–3^, assisting in or even automating the creation of literature reviews^4^, enhancing academic writing and referencing^5,6^, automatically formulating novel hypotheses^7,8^, developing specialized conversational agents^9,10^, and much more. Nonetheless, a significant challenge remains: processing the vast quantity of scientific papers, which continues to expand at a rate of approximately 2.5 million new articles each year^11^. The large number of research articles, often found in hard-to-parse PDF files or, at best, as unstructured plain text, poses significant challenges for AI technologies^12^. This issue limits the effectiveness of existing tools in fully understanding and exploring the literature^13^. As a result, current search engines are limited, mainly performing simple searches based on keywords or the semantic similarity of a query with titles and abstracts. Searching and analyzing this extensive body of text is a significant challenge even for advanced systems based on the latest Large Language Models (LLM)^14–18^. For instance, a recent study by Gan *et al*.^19^ discusses the limitations of LLMs in their ability to effectively identify and classify software and dataset mentions. Despite their impressive capabilities in natural language processing, these models struggle to achieve the level of accuracy necessary for such tasks, particularly when it comes to meeting the high standards of precision and recall demanded within the scholarly domain. Even the adoption of techniques such as Retrieval-Augmented Generation (RAG)^20^ does not solve this problem due to the complexity involved in searching and summarizing such an extensive collection of documents. The capability of effectively interpreting and answering natural language questions about a single 15-page paper, a task currently undertaken by several LLM-based services^4^, does not extend to understanding the key entities, narratives, and concepts of a research field represented by millions of such papers. This issue is very evident in the computer science domain, where it is notably difficult to navigate and assess the variety of methods and datasets that are continuously produced by the community. For instance, numerous methods that are described as state-of-the-art have reproducibility challenges and, when evaluated by external parties, might not outperform basic techniques^21,22^.

To address these challenges, a knowledge-centric paradigm has been suggested^23,24^. This approach involves creating a structured, interconnected, and formal representation of research publications to improve the capabilities of AI systems to conduct complex and extensive analyses of scientific texts.

In recent years, the research community has successfully used knowledge graphs (KGs) for creating semantically enriched representations of data in various domains, including scholarly knowledge. KGs are data structures that describe the key entities in a domain and their relationships, presenting information in a format accessible and interpretable by both machines and humans^25^. The relationship between two entities is typically formalized as a triple in the format of <subject, predicate, object> following the Resource Description Framework (RDF) as standard model (e.g., <sentiment analysis, uses, deep learning classifier> or <cloud computing, includes, virtualization security>). KGs are recognized for their ability to organize data in a structured and semantically meaningful manner, offering significant support to AI systems across multiple domains such as medicine, research, education, robotics, manufacturing, and social media, among others^26^. They are often realized by using Semantic Web technologies, such as RDF and the Web Ontology Language (OWL)^27^, that allow human experts to verify, curate, and correct both the data and their ontological schema. Additionally, KGs can be enhanced and refined using link prediction techniques, which are designed to discover new relationships between entities within a domain^28,29^. Prominent instances of KGs include DBpedia^30^, Google Knowledge Graph, BabelNet, and YAGO.

In the scientific field, the community has advanced this paradigm by developing bibliographic repositories linked to the Linked Data Cloud^31^, generating representations of scientific workflows^32^, managing pieces of information in nano-publications^33,34^, and designing a variety of vocabularies and ontologies to annotate scholarly data^35,36^. A recent survey^37^ identified 45 knowledge organization systems, including thesauri, taxonomies, and ontologies, which describe research fields at various levels of granularity.

In recent years, we have seen the release of KGs that describe the metadata of research publications, such as SemOpenAlex^38^, as well as KGs that focus on the content of these publications and describe the key entities and concepts such as topics, methods, and tasks. The content-focused KGs are either created through crowdsourcing (e.g., ORKG^39^, Nanopublications^34^) or generated automatically (e.g., AI-KG^40^, CS-KG^41^, SoftwareKG^42^). Crowdsourced KGs suffer from their limited coverage in terms of the number of articles because they rely on human experts to extract information from scholarly literature^34,39^ or focus on very specific domains such as intrusion detection^43^ or computational linguistics^44^. To tackle this challenge, in 2020, we introduced the Artificial Intelligence Knowledge Graph (AI-KG)^40^. This resource is the first automatically generated large-scale KG of artificial intelligence, including 1.2 million statements about over 800 thousand research entities. However, user feedback has highlighted some limitations of AI-KG: its coverage is restricted to 330,000 research papers, the semantic depth of the relationships between entities is relatively shallow, and its scope is confined exclusively to the domain of artificial intelligence.

In 2022, we released the Computer Science Knowledge Graph (CS-KG)^41^, building upon our previous efforts. This enhanced KG has a broader array of relationships between research entities and covers approximately 6.7 million research publications across a wide range of computer science subdomains. CS-KG has represented a significant step forward in addressing previous challenges, but it also faces a few notable limitations. First, CS-KG lacks time-related information for its entities, which is essential for analyzing trends in the adoption and evolution of various methods and materials. Second, it only includes research papers that are cited at least once, thereby excluding some of the most recent publications. Finally, CS-KG is based on the Microsoft Academic Graph (MAG), a resource that has since been discontinued. This means that this version of CS-KG cannot be updated or easily linked to a current and well-recognized metadata catalog.

In 2021, to fill the gap left by the discontinuation of MAG, the OpenAlex^45^ project started. OpenAlex^45^ is a free and open-access catalog of works, authors, venues, and institutions about the scholarly domain. One of its primary objectives is to sustain scientific initiatives previously reliant on MAG^40,46–49^. At the time of writing, OpenAlex covers more than 250M works including scientific publications in conferences or journals, about 90M disambiguate authors, and more than 100K institutions. While it has already been transformed into a KG^38^, there is no existing work or resource that provides a formal representation of the content of the publications.

In this paper, we present the newest version of CS-KG^50^ (CS-KG 2.0), which covers all the papers within the computer science domain indexed within the OpenAlex catalog from 2010 to 2022. Notably, OpenAlex is completely open-source and freely available under the CC0 license. Since CS-KG 2.0^50^ provides the unique identifiers of the papers used in its construction, users can access all relevant metadata via the OpenAlex API or its data dumps. The novel version of CS-KG 2.0^50^ includes over 1 billion RDF triples and approximately 24 million research entities categorized into types such as *Task*, *Method*, *Metric*, *Material*, and *OtherEntity*. It also includes 67 million statements that detail the relationships among these entities. Consequently, this version is more than 50 times larger than its predecessor. Compared to earlier versions, CS-KG^50^ 2.0 introduces significant enhancements, including i) the incorporation of temporal data linked with entities and statements, which facilitates the analysis of research trends over time, and ii) the provision of supplementary contextual information. This extra information enhances the comprehension and analysis of the data contained within its statements, highlighting, for instance, the co-occurrence patterns among entities and statements. This initiative aims to support a range of intelligent services, including advanced search, question-answering systems, conversational agents, article recommendation, trend forecasting, hypothesis generation, and many others.

In conjunction with CS-KG^50^ 2.0, we also release three extensive benchmarks for the evaluation of link prediction algorithms: CSKG-2M, CSKG-490K, and CSKG-132K. These new resources, derived from CS-KG 2.0, are significantly larger than established benchmarks in this field, such as WN18 and FB15k^51^.

CS-KG^50^ 2.0 is licensed under a Creative Commons Attribution 4.0 International License (CC BY 4.0). It is available as a dump in RDF and CSV format at 10.5281/zenodo.14167681. It can also be queried via a SPARQL endpoint at https://w3id.org/cskg/sparql. In all subsequent sections of the paper, we utilize CS-KG as the singular reference for CS-KG^50^ 2.0.

## Methods

### Knowledge Graph Generation

**Fig. 1:**
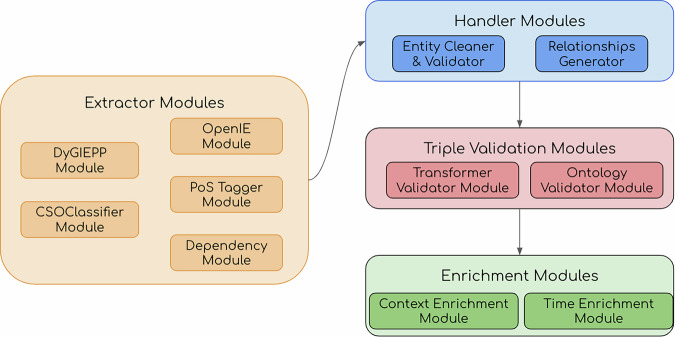
The modules used to create our resource from the SCICERO^52^ pipeline.

The resource released in this paper is built using an automatic pipeline named *SCICERO* presented in Dessí *et al*.^52^ Fig. 1 reports an overview of the architecture. In this section, we briefly describe the main components and provide the scientific rationale behind each. Specifically, the approach is composed of several modules that can be grouped into four categories: i) *extraction modules*, which parse text from the title and abstract of the research papers, ii) *handler modules*, which clean and normalize the extracted data, iii) *validation modules*, which discard noisy elements from the final set of generated triples, and iv) *enrichment modules*, which add additional contextual information to the extracted statements.

#### Extractor Modules

The current version of the *SCICERO* pipeline uses five extraction modules: DyGIEpp, CSO Classifier, OpenIE, PoS Tagger, and Dependency Module. We describe each of them in the following.

The **DyGIEpp Module** leverages the tool described in Wadden *et al*.^53^, which adopts a BERT-based model pre-trained on the *SciERC*^54^ dataset. This module extracts 6 types of entity (i.e., *Method*, *Task*, *Metric*, *Material*, *Other-Scientific-Term*, and *Generic*) and 7 types of relations between entities (i.e., *Used-for*, *Hyponym-Of*, *Compare*, *Part-of*, *Conjunction*, *Feature-of*, *Evaluate-for*). CS-KG^50^ does not differentiate between Other-Scientific-Term and Generic and thus, the module merges them in the *OtherEntity* entity type. DyGIEpp employs a feed-forward neural network to process textual span representations, generating two scores. The first score indicates the likelihood of a given text being a research entity belonging to one of the predefined types. The second score represents the probability of a predefined relationship existing between two identified entities, considering the context in the text. A softmax function is applied to distinguish entity types and relationships. For instance, in the sentence “*Training time for large language models is usually lengthy*.”, the text span “training time” is recognized as a research entity of type *Metric* with a softmax value of 0.99, while “large language models” is identified as an entity of type *Method* with a softmax value of 1.0. The relation *Feature-of* is identified with a softmax value of 0.54 in the generated triple <training time, Feature-of, large language models>. The relations extracted by DyGIEpp are then transformed into a set of predicates using mapping rules that are publicly available online (https://github.com/danilo-dessi/SKG-pipeline/blob/main/resources/SKG-dygiepp-Mapping.csv). For example, the triple <training time, Feature-of, large language models> is transformed into <large language models, includes, training time>.

The **CSO Classifier Module**^36^, based on the Computer Science Ontology (CSO)^55^, identifies research topics within textual content. Specifically, it evaluates whether a given text span aligns with one of the over 14,000 topics defined in the CSO.

For this purpose, it leverages two unsupervised components. The syntactic component identifies unigrams, bigrams, and trigrams using a rule-based method and employs the Levenshtein similarity to compare these n-grams with topics in the CSO. The semantic component utilizes a combination of part-of-speech tagging and similarity computed via a Word2Vec model to map a text span with topics in CSO.

The output of this module is the set of identified entities.

The **OpenIE Module** extracts triples using the Stanford Core NLP^56^ suite and its OpenIE annotator^57^. It operates as follows. First, it creates clauses, i.e., groups of words containing a subject-noun and a verb which are identified by traversing the parsing tree of the input text. These clauses are then shortened, creating concise fragments, which are subsequently transformed into triples. Finally, the module selects only triples in which both subjects and objects overlap with the entities identified by the DyGIEpp and CSO classifier modules.

**The PoS Tagger Module** is designed for extracting triples and is based on the *part of speech* component from the Stanford Core NLP suite^56^. This module identifies verbs located between pairs of entities that have already been extracted. Subsequently, it forms triples in the form <entity_1_, verb, entity_2_ > . To minimize the inclusion of irrelevant or weakly associated relationships, the module applies a filtering mechanism to exclude entity pairs that are too distant within the text. Specifically, it considers only entity pairs separated by a number of tokens within a predefined window size. This approach effectively reduces the likelihood of capturing noisy or spurious relationships between entities, ensuring a focus on those with meaningful or direct relevance.

**The Dependency Module** extracts triples by extending the functionality of the Stanford Core NLP Dependency Parser. It derives meaningful triples by leveraging a set of predefined paths within the dependency trees of a sentence. The module is designed to extract relationships based on twelve paths (https://github.com/danilo-dessi/SKG-pipeline/blob/main/resources/path.txt) that were manually designed by analyzing the frequency and quality of generated triples^52^. These paths represent specific patterns in the dependency structures of sentences that frequently capture meaningful relationships between entities. To identify these paths, dependency trees from a sample of scientific papers were analyzed, with a focus on paths containing verbs that consistently connect pairs of research entities. The paths were evaluated based on the quality and frequency of the resulting triples, with only those generating over 60% correct triples being retained. This process yielded twelve highly effective paths for capturing high-quality relationships.

#### Handler Modules

**Entity Cleaner and Validator**. This module performs several tasks to filter and improve the quality of the extracted entities: (i) it lemmatizes all entities to map singular and plural forms to the same text representation; (ii) it solves acronyms by leveraging their common placement in brackets near relevant entities; (iii) it discards entities included in a manually curated denylist; and (iv) it identifies and removes generic entities by assessing their information content score, which quantifies the specificity of words based on the *WordNet* taxonomy (https://wordnet.princeton.edu/). To ensure essential entities for the domain are retained, the module also utilizes a white list of research entities, derived from the *Fields of Study* provided by the original source.

**Relationship Integrator**. The sets of triples extracted by the DyGIEpp Module, OpenIE Module, PoS Tagger Module, and Dependency Module, namely *T*_*Dy*_, *T*_*OIE*_, *T*_*PoS*_, and *T*_*Dep*_ may contain redundant triples that use different predicates to express the same meaning (e.g., *includes*, *embeds*, *contains*). To address this issue, we associate similar verbs with an exemplary predicate. This mapping schema is built by combining relation clustering using the word2vecembedding model^58^ with the VerbNet (https://verbs.colorado.edu/verbnet/) taxonomy as described in Dessí *et al*.^52^ VerbNet provides a comprehensive taxonomy of English verbs organized into classes, where verbs within the same class share both syntactic and semantic coherence. It allows the creation of domain-specific taxonomies while maintaining a core representation of verbs based on their semantics in more general contexts. We used it to construct the CS-KG^50^ ontology (https://scholkg.kmi.open.ac.uk/cskg/ontology), which describes a set of 39 predicates mapped to 464 verbs. All relations in the sets *T*_*Dy*_, *T*_*OIE*_, *T*_*PoS*_, and *T*_*Dep*_, are mapped using this schema. For instance, two triples sharing the same entities, such as <a, embeds, b> and <a, contains, b>, are consolidated into a single triple <a, includes, b>, where *embeds* and *contains* are mapped to *includes*. Following the mapping of all relations in the sets *T*_*Dy*_, *T*_*OIE*_, *T*_*PoS*_, and *T*_*Dep*_, the module produces a unique set of triples *T*.

#### Triple Validation Modules

**The Ontology Validator Module**. This component uses the CS-KG^50^ ontology to consolidate triples generated by the various tools and discards those that do not adhere to the specified domain and range constraints of the defined object properties. All triples in the set *T* that are compatible with the CS-KG^50^ ontology are then represented by RDF statements in accordance with the ontology. The entity types returned by the DyGIEpp tool are aligned with relevant classes in the ontology. Specifically, entities such as methods, tasks, materials, and metrics are mapped to corresponding classes in the ontology (e.g., the type *material* is mapped to the class *cskg-ont:Material*), while *other scientific terms* and *generic* entity types are mapped to *cskg-ont:OtherEntity*. The predicates are also mapped to the object properties of the ontology. For example, the triple <cskg:dimensionality_reduction, uses, cskg:similarity_metric>, where *cskg:similarity_metric* is a *cskg-ont:Metric* is transformed into <cskg:dimensionality_reduction, cskg-ont:usesMetric, cskg:similarity_metric>. During this phase, triples that do not adhere to the semantics of the ontology are discarded. For instance, the triple <cskg:textbook_dataset, uses, cskg:spatial_prediction>, where *cskg:textbook_dataset* is a *cskg-ont:Material* and *cskg:spatial_prediction* is a *cskg-ont:Task*, is discarded because the class *cskg-ont:Material* is not in the domain of the property *cskg-ont:usesTask*. In simpler terms, a material cannot utilize a task according to the CS-KG^50^ ontology.

**Transformer Validator Module**. A triple derived from several articles is generally of reliable quality, given the low likelihood of extracting the same erroneous information from multiple textual sources. Conversely, triples found in only one or a few papers tend to be more prone to noise. To discern between reliable and possibly problematic triples, we introduce the concept of *support* denoting the number of papers associated with a specific triple. However, discarding all uncertain triples could lead to significant information loss, since many of these triples may still be valid. Consequently, we employ a machine learning classifier to determine which low-supported triples exhibit high-quality characteristics and can thus be integrated into the KG. First, we partition the set of all triples, denoted as *T*, into two groups: *T*_*reliable*_, which includes triples with a support ≥3, and *T*_*uncertain*_, which includes all other triples. Subsequently, the set *T*_*reliable*_ is utilized to fine-tune a SciBERT^59^ classifier on a binary classification task. This classifier implements a function *θ*: <s,p,o> → 0, 1 that when provided with an input triple <s,p,o>, predicts 1 if the triple is accurate and can be included in the KG, and 0 if the triple should be discarded. For the fine-tuning phase, we provide both positive and negative examples to the classifier. Positive triples correspond to the set *T*_*reliable*_ whereas negative triples are generated by corrupting each triple *t* ∈ *T*_*reliable*_ with a triple $t{\prime}$ where $t{\prime} \notin T$, i.e., $t{\prime}$ is not triple that is extracted by our approach. Triples corruption involves replacing either the head or the tail with a randomly chosen entity, resulting in the set of negative triples denoted as *T*_*negative*_. Consequently, the combined set *T*_*reliable*_ ∪ *T*_*negative*_ is employed to fine-tune the model. The underlying rationale of this methodology is to leverage the classifier’s capability to identify high-quality triples within the set *T*_*uncertain*_, aligning with the characteristics of triples in *T*_*reliable*_. The set of triples for which the classifier predicts 1 is denoted as *T*_*consistent*_.

Finally, the triples in sets *T*_*reliable*_ and *T*_*consistent*_, along with all associated information, are converted into RDF statements through reification. Reification is a data modeling solution used to provide additional information regarding specific triples within an RDF graph^60^. It is commonly utilized to append metadata to triples, such as sources, confidence levels, and context-related annotations^61^. In the CS-KG^50^ pipeline, reification entails the creation of a new RDF statement for each triple. This statement describes the original triple through the relations *rdf:subject*, *rdf:predicate*, and *rdf:object*. Additional properties are incorporated via different relations. For example, the relation *provo:wasDerivedFrom* is employed to list the academic papers that were used to generate the triple. The enrichment modules, described in the next subsection, expand this representation by integrating more metadata. An illustration of a fully reified statement is presented in the Data Records Section.

#### Enrichment Modules

This section describes the modules utilized to enhance the extracted statements with additional information.

**Context Enrichment Module**. This module is designed to materialize additional information that can be inferred from the generated statements. The objective is to analyze each statement to identify the statements and entities that most frequently co-occur with it. This process provides deep insights into the contextual framework within which a statement is situated. The co-occurrences are computed as in the following. First, for each statement *s*, the module retrieves the list of papers from where it was extracted, let us refer to it as *P*. Next, for each paper *p* ∈ *P*, it extracts all entities and statements linked to *p* and calculates their respective frequencies of occurrence. For example, if the statement *s* is extracted from the papers {*p*_1_, *p*_2_, *p*_3_} where *p*_1_ contains the entities {*e*_1_, *e*_2_, *e*_3_}, *p*_2_ contains the entities {*e*_2_, *e*_3_, *e*_4_}, and *p*_3_ contains {*e*_2_, *e*_3_, *e*_5_}, then the statement *s* is associated to the entities *e*_2_ and *e*_3_ with number of occurrences equal to 3 and to *e*_1_, *e*_4_, and *e*_5_ with number of occurrences equal to 1. The same process is applied to compute the co-occurrent statements. This process is applied only to statements that are associated with at least 5 research papers, thus ensuring sufficiently robust association with the literature. The output of the module is a set of contextual entities and statements for each statement.

**Time Enrichment Module**. This module is engineered to augment statements and entities with temporal data, derived from the publication years of the source papers from which they are extracted. More specifically, it determines the annual frequency of each entity and statement by quantifying their occurrences in the papers for each respective year. For example, let two statements be *s*_1_ and *s*_2_ describing the triples <s,p,o> and <s,p',o'>, respectively; <s,p,o> appears in the papers *p*_1_, *p*_2_, and *p*_3_ while <s,p',o'> appears in *p*_3_ and *p*_4_. Let us also assume that *p*_1_ and *p*_3_ are published in the year 2015, and *p*_2_ in the year 2017, and *p*_4_ in the year 2018. Then, this module associates *s* to the frequencies {2015: 2, 2017: 1, 2018: 1}, the frequencies {2015: 2, 2017: 1} to *o*, and {2015: 1, 2018: 1} to $o{\prime}$. Also, it associates {2015: 2, 2017: 1} to <s,p,o>, and {2015: 1, 2018: 1} to <s,p',o'>.

## Data Records

### CS-KG

CS-KG^50^ is available in Zenodo with reference number *14167682* at 10.5281/zenodo.14167681. The current version contains 67*M* statements describing 24M research entities extracted from 14.5M research publications in computer science in the period 2010-2022. Its data schema is formalized by the CS-KG^50^ ontology, available at https://scholkg.kmi.open.ac.uk/cskg/ontology. Detailed documentation is provided at https://w3id.org/cskg. This OWL ontology is built upon SKOS (http://www.w3.org/2004/02/skos/core#) and PROV-O (http://www.w3.org/ns/prov#) vocabularies. It employs https://w3id.org/cskg/ontology# (prefix *cskg-ont*) and https://w3id.org/cskg/resource/ (prefix *cskg*) to denote the schema vocabulary and the instances, respectively. The ontology defines 219 object properties, i.e., relations that can exist between two entities, such as *cskg-ont:usesMethod* and *cskg-ont:solvesTask*, among five distinct entity types: *cskg-ont:Task*, *cskg-ont:Method*, *cskgont:Material*, *cskg-ont:Metric*, and *cskg-ont:OtherEntity*.

CS-KG^50^ is designed to systematically represent information extracted from research publications in the form of structured statements. Each statement within CS-KG^50^ describes a triple that defines the relationship between two entities as identified within a set of research papers.

For instance, the triple <internet_of_thing, includesMethod, wireless_protocol> is encapsulated by the following statement:

cskg:statement_129110 a cskg-ont:Statement,

provo:Entity;

rdf:subject cskg:internet_of_thing;

rdf:predicate cskg-ont:usesMethod;

rdf:object cskg:wireless_protocol;

cskg-ont:hasSupport "8"^^rdfs:Literal;

provo:wasDerivedFrom cskg:W4200396539,

cskg:W3122863749,

cskg:W4285125700,

cskg:W2734803290,

cskg:W2736432465,

cskg:W1998514859,

cskg:W2527500525,

cskg:W3123263801;

provo:wasGeneratedBy cskg:dependency_tagger,

cskg:dygiepp;

cskg-ont:hasTimeObservation cskg:observation_statement_129110_2015,

cskg:observation_statement_129110_2016,

cskg:observation_statement_129110_2017,

cskg:observation_statement_129110_2019,

cskg:observation_statement_129110_2021,

cskg:observation_statement_129110_2022.

In the above statement, the reader can observe the following properties:

- *rdf:subject* and *rdf:object*, describing the entities of the triple.
- *rdf:predicate* that describes the relationship between subject and object.
- *cskg-ont:hasSupport*, which links the statement to the number of papers the triple is extracted from. The support score can be utilized to select subsets of the knowledge graph that are more trustworthy and, therefore, have higher significant evidence.
- *provo:wasDerivedFrom*, which refers to the IDs of research papers from OpenAlex from where the triple is extracted.
- *provo:wasGeneratedBy*, which refers to which modules of the generative pipeline detected the triple.
- *cskg-ont:hasTimeObservation*, which links the statement to sub-statements that describe the number of appearances for each year in which the underlying statement is detected. An example of time observation for the year 2021 of the statement above is:

cskg:observation_statement_129110_2021 a cskg-ont:TimeObservation;

cskg-ont:year "2021"^^rdfs:Literal;

cskg-ont:frequency "2"^^rdfs:Literal.

Supplementary to the previously mentioned details, contextual information is incorporated to delineate the co-occurrences of CS-KG^50^ statements with other entities and statements. Contextual information is generated for statements that are supported by at least 5 papers. To ensure the focus on pertinent contexts, only co-occurrences whose score is equal to or greater than 2 are inferred. For example, the statement above is contextualized with other entities as follows:

cskg:statement_129110 cskg-ont:hasContextEntityObservation cskg:entity_observation_2309882.

cskg:statement_129110 cskg-ont:hasContextEntityObservation cskg:entity_observation_2309883.

…

cskg:statement_129110 cskg-ont:hasContextEntityObservation cskg:entity_observation_2309890.

cskg:statement_129110 cskg-ont:hasContextEntityObservation cskg:entity_observation_2309891.

cskg:statement_129110 cskg-ont:hasContextEntityObservation cskg:entity_observation_2309892.

Each observation corresponds to an entity and its co-occurrence with the statement as in the example below:

cskg:entity_observation_2309892 a cskg-ont:ContextEntityObservation;

cskg-ont:relatedEntity cskg:wireless_technology;

cskg-ont:co-occurrence "2"^^rdfs:Literal.

Furthermore, for each entity, CS-KG^50^ also provides information about their distribution over the years. For example, for the entity *cskg:internet_of_thing*, CS-KG^50^ contains the following linkages to time observations:

cskg:internet_of_thing cskg-ont:hasTimeObservation cskg:entity_time_observation_17565238.

cskg:internet_of_thing cskg-ont:hasTimeObservation cskg:entity_time_observation_17565239.

…

cskg:internet_of_thing cskg-ont:hasTimeObservation cskg:entity_time_observation_17565248.

cskg:internet_of_thing cskg-ont:hasTimeObservation cskg:entity_time_observation_17565249.

cskg:internet_of_thing cskg-ont:hasTimeObservation cskg:entity_time_observation_17565250.

Similarly to the statements, time observations associated with an entity hold information about the year and the number of appearances. For example the time observation *cskg:entity_time_observation_17565238* in CS-KG^50^ is as follows:

cskg:entity_time_observation_17565238 a cskg-ont:TimeObservation;

cskg-ont:year "2010"^^rdfs:Literal;

cskg-ont:frequency "112"^^rdfs:Literal.

This indicates that the entity *cskg:internet_of_thing* appeared 112 times in the abstracts of research papers published in 2010.

Finally, CS-KG^50^ provides links between its entities and corresponding entities in DBpedia and Wikidata, facilitating the integration of CS-KG^50^ with some of the largest KGs available. For example the entity *cskg:sentiment_analysis* is linked as follows:

@prefix dbpedia:http://dbpedia.org/resource/.

@prefix wikidata: http://www.wikidata.org/entity/.

cskg:sentiment_analysis owl:sameAs dbpedia:Sentiment_analysis.

cskg:sentiment_analysis owl:sameAs wikidata:Q2271421.

All the information contained in the CS-KG^50^ can be accessed through a dedicated SPARQL endpoint at https://w3id.org/cskg/sparql or downloaded as a dump. We provide the data in RDF and CSV formats at 10.5281/zenodo.14167681.

The dump contains 3 main directories *rdf/*, *csv/*, and *benchmark/*. The *rdf/* and *csv/* directories provide the same data in two different formats, each containing the following set of files:

- The files *cskg_data_N.ttl* (*cskg_data_N.csv*), where N denotes the file split number, contain the following properties: the subject, predicate, and object of the triples, their support, the paper from where the triple was derived, the tools used to generate the triple, the time observations, and the triple ID.
- The files *dbpedia.ttl* (*dbpedia.csv*) and *wikidata.ttl* (*wikidata.csv*) map the entities to their corresponding ones in DBpedia and Wikidata.
- The file *paper_info.ttl* (*paper_info.csv*) includes the ID, year, and DOI (if available) of the research papers.
- The file *entity_type.ttl* (*entity_type.csv*) associate entities to their types.
- The file *cskg_entity_year_distribution.ttl* (*cskg_entity_year_distribution.csv*) contains the yearly distribution of entities.
- The files *time_cskg_data_N.ttl* include the statement time observations for the RDF dump. This same information is included in the files *cskg_data_N.csv* for the CSV dump. This difference arises from the differences in data formats and reflects the most convenient approach for handling each format.
- The files *stm_entity_context.ttl* (*stm_entity_context.csv*) and *stm_statements_context.ttl* (*stm_statements_context.csv*) contain the contextual information about the entities and statements, respectively.
- The files *prefix.ttl* and *cskg_onto_v2.ttl* are required to import the RDF dump into SPARQL endpoints and Semantic Web-based tools.

In addition to CS-KG, we provide three benchmarks for link prediction, available in the directory *benchmark*: CSKG-2M, CSKG-490K, and CSKG-132K. Each benchmark includes the following files:

- *entities.txt* contains the entities and their corresponding IDs within the benchmark.
- *relations.txt* includes the relations and their corresponding IDs within the benchmark.
- *triples.txt* lists all the triples in the benchmark, encoded using the IDs from *entities.txt* and *relations.txt*.
- *train.txt*, *valid.txt*, and *test.txt* contain subsets of triples extracted from *triples.txt* that are designated as the training, validation, and test sets, respectively.

### CS-KG Statistics

In this section, we explore various analytical views on CS-KG^50^ to illustrate its scale and versatility.

**Fig. 2:**
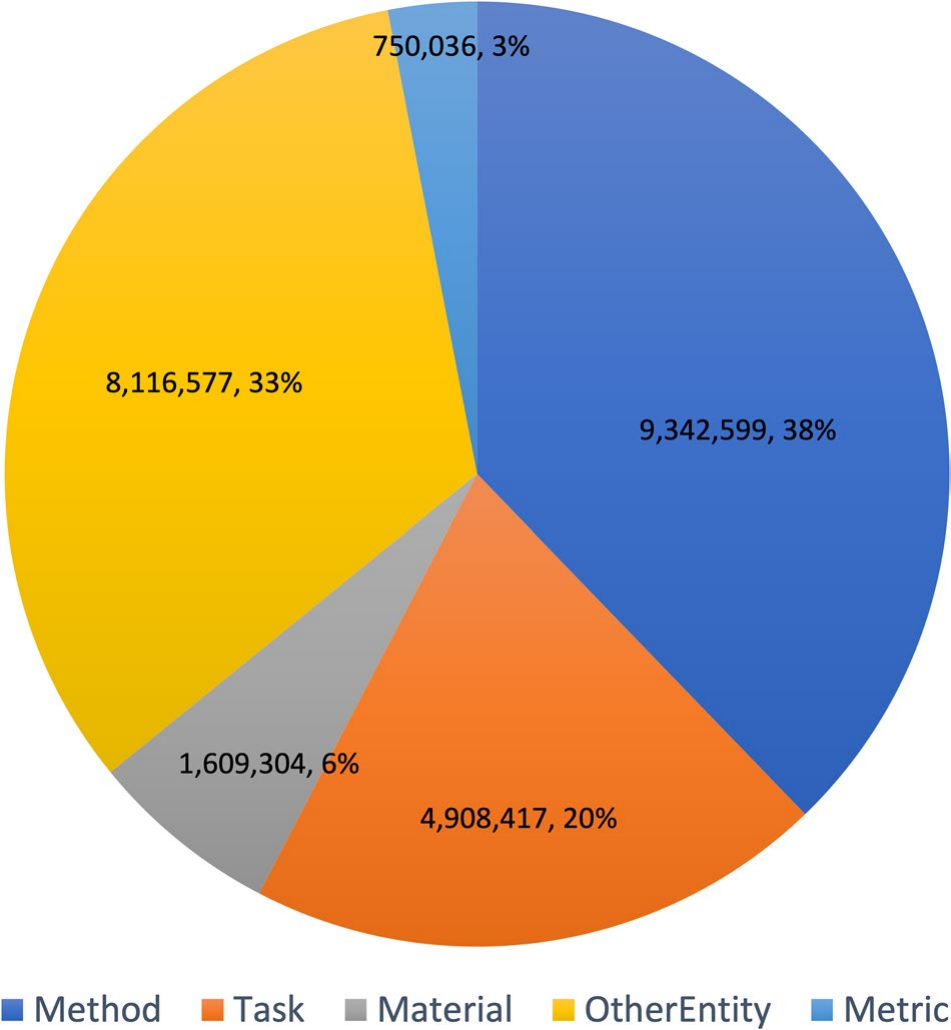
The distribution of the entities across the classes Method, Task, Material, Metric, and OtherEntity.

Figure 2 reports the number of entities for each type. *Method* and *OtherEntity* are the most frequent types, followed by *Task*, *Material*, and *Metric*.

**Fig. 3:**
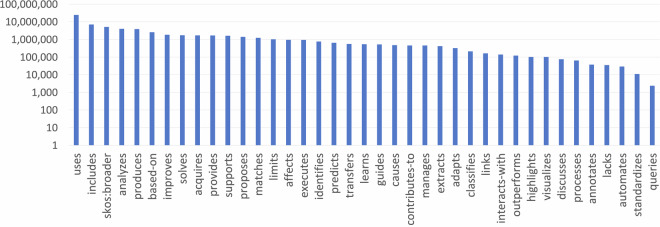
The distribution in logarithmic scale of the relationships over the statements based on their verbs.

Figure 3 illustrates the distribution of statements based on the 39 main predicates that were used to produce the object properties (plus the *skos:broader* relation from the DyGIEpp module). The most common predicates are *uses*, *includes*, *skos:broader*, *analyses*, and *produces*.

**Fig. 4:**
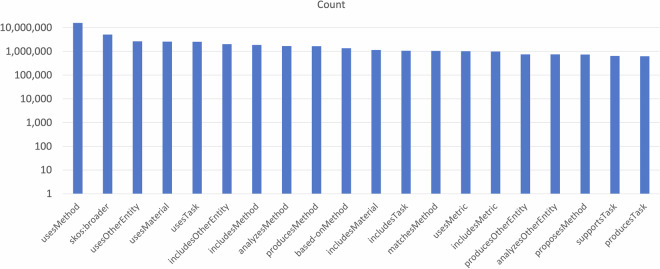
The distribution of the 20 most frequent relationships over the statements based on their verbs and classes.

In Fig. 4, we report the distribution of the statements on the 20 most frequent object properties. As earlier noted, these properties are relations that incorporate both the predicates and entity types. This figure demonstrates the variety of statements in the CS-KG^50^, showcasing a wide range of diverse predicates.

**Fig. 5:**
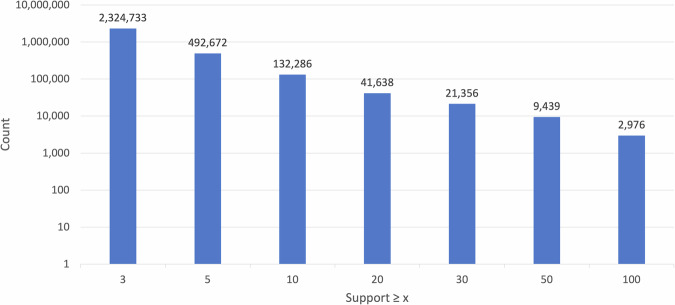
The distribution in logarithmic scale of the statements with support greater or equal to a threshold x.

Figure 5 depicts the distribution of CS-KG^50^ statements across different support levels. The visualization highlights that CS-KG^50^ offers a substantial number of well-supported statements (support ≥5) and thus can reliably support knowledge-driven applications.

Finally, Table 1 presents a comparison between the latest version of CS-KG^50^ and its earlier version (CS-KG 1.0), as well as its precursor that is exclusively focused on the domain of artificial intelligence (AI-KG). This comparison underscores the significant advancements made in CS-KG^50^ over CS-KG 1.0, particularly in terms of the completeness of scientific information. Notably, CS-KG^50^ has seen considerable increases in the number of entities (from 10M to 24.7M), statements (from 41M to 67.5M), covered papers (from 6.7M to 14.5M), and links to external knowledge bases (from 58K to 229K).

**Table 1:** Comparison between CS-KG^50^, CS-KG 1.0, and AI-KG graphs.

Feature	CS-KG^50^	CS-KG 1.0^41^	AI-KG^40^
Number of Entities	24.7M	10M	820K
Number of Statements	67.5M	41M	1.2M
Number of covered Scientific Papers	14.5M	6.7M	333K
Number of Ontology Axioms	2,275	2,213	321
Number of Object Properties	219	179	27
Links to DBpedia	203K	31K	0
Links to Wikidata	26K	27K	19K
Multiple relationships between two Entities	yes	yes	no
Temporal Information	yes	no	no
Contextual Information	yes	no	no

## Technical Validation

In this section, we present a formal evaluation of the quality of the extracted triples within CS-KG^50^. We also explore the performance of various state-of-the-art algorithms for link prediction on a new benchmark based on CS-KG^50^.

### Evaluation of the Triples Quality

We evaluate the quality of the triples in CS-KG^50^ by manually assessing a subset of 3, 600 triples. For this purpose, we have selected:

- 900 triples sourced from CS-KG^50^ with a support of 3 or more. These triples are considered reliable as they have been identified in at least three distinct research papers.
- 900 triples from CS-KG^50^ with a support lower than 3. These triples feature information that is less common in the literature, which could make them noisier.
- 800 triples discarded by the Ontology Validator Module, indicating information that conflicts with the CS-KG^50^ ontology.
- 800 triples discarded by the Transformer Validator Module. These triples include scientific assertions that were deemed potentially inaccurate or not aligned with the high-quality triples utilized to train the model.
- 200 triples randomly generated by corrupting triples from CS-KG^50^. These triples contain mostly erroneous facts.

The selection of triples is designed to ensure that either the subject or the object is relevant to one of three specific subfields of computer science: Natural Language Processing, Machine Learning, or Information Retrieval. This is achieved by curating a list of pertinent topics from the Computer Science Ontology. The list of topics for the evaluation is available at https://github.com/danilo-dessi/SKG-pipeline/blob/main/resources/cso_topics.txt.

Next, three senior researchers with strong expertise in the relevant topics have evaluated the 3,600 triples. They have assigned a value of 1 whenever a triple is correct, meaning it is corroborated by the literature, and 0 otherwise. They have been also permitted to use online resources to verify whether a triple aligns with established scientific research. The agreement between annotators, as measured by Fleiss’ kappa^62^, is 0.61, indicating a moderate level of agreement. We have then created a gold standard based on the majority vote.

To assess the performance of the information extraction method, we use the resulting gold standard to compare: 1) all extracted triples without any validation (row *Extractor & Handler Modules*), 2) only the triples validated by the Ontology Validator Module, 3) only the triples validated by the Transformer Validator Module, 4) the final triples produced by the full pipeline, validated by all the validators. We evaluate the quality of the triples using precision, recall, and F-measure.

Table 2 reports the result of the evaluation. The raw triples (first row), i.e., triples extracted without any further refinement and validation process, exhibit high recall but are quite noisy, as reflected in their low precision (47%). This suggests that while combining multiple tools provides extensive coverage of triples relevant to the computer science domain, it also introduces a considerable amount of incorrect or misleading information. The Ontology Validator Module increases precision by 10% while maintaining good recall. Similarly, the Transformer Validator Module increases precision by about 7%. The final version of the pipeline achieves a precision of 72% and a recall of 79%, resulting in an F-measure of 75%.

**Table 2:** Evaluation of triple sets generated by pipeline sub-modules.

Pipeline Modules	Precision	Recall	F-measure
Extractor & Handler Modules	0.47	**0.98**	0.64
Extractor & Handler Modules + Ontology Validator	0.57	0.91	0.70
Extractor & Handler Modules + Transformer Validator	0.54	0.86	0.66
Extractor & Handler Modules + Ontology Validator + Transformer Validator (**Full method**)	**0.72**	0.79	**0.75**

As discussed earlier, the accuracy of the triples is highly dependent on their support, defined by the number of associated papers. For instance, the triples supported by more than five papers yield a precision of 77% while triples with support greater than ten achieve a precision of over 91%. Therefore, studies or tools requiring greater precision can consider subsets of CS-KG^50^ that contain only triples with high support.

In summary, the evaluation reveals two primary insights. First, while the extraction method can generate a detailed representation of the field, it may also result in some inaccurate and irrelevant information. Second, the techniques used to validate the data, whether based on their alignment with highly supported triples (as in the Transformer Validator Module) or adherence to domain ontology (as in the Ontology Validator Module), can alleviate this issue and significantly improve the quality of the resulting triples.

### Link Prediction Benchmark

We are also releasing three benchmarks for link prediction based on CS-KG^50^: CSKG-2M, CSKG-490K, and CSKG-132K.

Link prediction is a widely studied task in machine learning that involves predicting the existence (or likelihood) of (typed) edges in a graph^63^. This task gains particular importance in the context of KGs, where it is also known as *knowledge graph completion*, due to its role in improving data quality by addressing data incompleteness and correcting erroneous relationships^64^. The purpose of these benchmarks is twofold: 1) to demonstrate how the triples in the CS-KG^50^ can serve as a knowledge base for machine learning techniques, and 2) to provide the research community with large-scale resources for evaluating and comparing link prediction approaches. Specifically, the link prediction benchmarks commonly used in the community, such as WN18^51^ and FB15k^51^, usually feature tens of thousands of triples. By current standards, these datasets are considered small. The resources we introduce aim to address this limitation by providing a large-scale benchmark.

The released benchmarks are as follows:

- **CSKG-2M** provides more than 2*M* triples from CS-KG^50^with a support ≥3. It contains about 1.5*M* triples for training, 278*K* triples for validation, and 465*K* triples for testing.
- **CSKG-490K** provides more than 490*K* triples with a support ≥5. It contains about 335*K* triples for training, 59*K* triples for validation, and 98*K* triples for testing.
- **CSKG-132K** provides 132*K* triples with a support ≥10. It contains about 89*K* triples for training, 15*K* triples for validation, and 26*K* triples for testing.

Therefore, CSKG-2M includes both CSKG-490K and CSKG-132K, and CSKG-490K includes CSKG-132K.

We have assessed on the new benchmarks four of the most prominent state-of-the-art solutions for link prediction: TransR^65^, ComplEx^66^, DistMul^67^, and RESCAL^68^. Although the research community continuously develops novel approaches for link prediction, these methods remain widely recognized, frequently included in state-of-the-art comparisons, and implemented in several software libraries, making them strong baselines for future research efforts.

Table 3 reports the performance of the four methods both on our benchmarks as well as on two widely recognized benchmarks in the field: FB15k, derived from Freebase, and WN18, derived from WordNet^51^. The three benchmarks derived from CS-KG^50^ are more challenging than existing benchmarks for two primary reasons. First, they include a wider selection of relations. Second, they offer a substantially larger volume of triples, testing the scalability of link prediction methods. Consequently, these benchmarks offer novel and more demanding resources to advance research in link prediction.

**Table 3:** Performance comparison of link prediction methods across the three novel benchmarks and two established benchmarks (FB15k and WN18).

Dataset	LP Method	MRR	MR	Hits@1	Hits@3	Hits@10
FB15k	TransR	0.670	59.99	0.585	0.728	0.808
FB15k	DistMul	0.696	61.43	0.586	0.782	0.873
FB15k	ComplEx	0.757	64.73	0.672	0.826	0.886
FB15k	RESCAL	0.661	124.5	0.589	0.704	0.787
WN18	TransR	0.609	432.8	0.452	0.736	0.850
WN18	DistMul	0.813	419.0	0.702	0.921	0.948
WN18	ComplEx	0.932	318.2	0.914	0.948	0.959
WN18	RESCAL	0.848	563.6	0.792	0.898	0.928
CS-KG-2M	TransR	0.456	150.83	0.391	0.491	0.574
CS-KG-2M	ComplEx	0.418	223.37	0.361	0.453	0.515
CS-KG-2M	DistMult	0.421	219.27	0.362	0.457	0.520
CS-KG-2M	RESCAL	0.375	160.11	0.301	0.412	0.509
CS-KG-490K	TransR	0.563	120.49	0.488	0.611	0.695
CS-KG-490K	ComplEx	0.469	133.20	0.389	0.517	0.610
CS-KG-490K	DistMult	0.470	128.39	0.388	0.520	0.617
CS-KG-490K	RESCAL	0.448	114.18	0.363	0.493	0.605
CS-KG-132K	TransR	0.518	123.61	0.425	0.579	0.685
CS-KG-132K	ComplEx	0.392	138.96	0.290	0.452	0.582
CS-KG-132K	DistMult	0.404	129.24	0.302	0.462	0.594
CS-KG-132K	RESCAL	0.385	118.18	0.294	0.431	0.557

## Usage Notes

Our dataset is provided in *Turtle* syntax for the RDF format and can be loaded within any triplestore supporting it, such as Openlink Virtuoso, Blazegraph, and GraphDB. We also provide all data in CSV format. Both the Turtle and CSV dumps are available at 10.5281/zenodo.14167681. Additionally, the resource can be accessed for exploration in a live triplestore available at https://w3id.org/cskg/sparql.

In the following, we showcase a series of exemplary queries that can be run on CS-KG^50^.

These queries should provide a starting point for facilitating the use of CS-KG^50^. It is important to recognize that these examples align with the Competency Questions commonly adopted by the semantic web and knowledge graphs community to evaluate the capabilities of a knowledge base. It is acknowledged that there may be additional use cases not addressed in this section.

For each sample query, we present the question in natural language and its corresponding SPARQL query. All SPARQL queries are prefaced with a standard header:

We list the exemplary queries in the following.

*1. Retrieve all statements about a specific entity (e.g., Wikipedia) that are supported by at least 10 articles*.

*2. Given a research entity (e.g., sentiment analysis) as the subject, retrieve the relevant statements ordered by support*.

*3. Given a material (e.g., Twitter), identify the tasks for which it was used and list the articles from which this information was derived*.

*4. Retrieve the statements derived from a paper with a specific ID (e.g., W2060391699) in the OpenAlex catalog*.

*5. Given a research entity (e.g., sentiment analysis), return all corresponding entities on external KGs*.

*6. List the entities that appeared in at least 100 triples extracted from papers in 2015*.

*7. List the statements with a frequency of at least 100 in the year 2020*.

*8. Return the frequency of an entity (e.g., machine learning) over the years*.

*9. Return the frequency over the years of a statement (e.g., <machine learning, usesMaterial, twitter>)*.

*10. Given a statement (e.g., <internet_of_things, usesMethod, wireless_protocol>), retrieve the top 20 entities that most frequently co-occur with it*.

*11. Given a statement (e.g., <internet_of_things, usesMethod, wireless_protocol>), retrieve the top 20 statements that most frequently co-occur with it*.

## Data Availability

The codebase and the documentation of the pipeline used to generate CS-KG^50^ can be found at: https://github.com/danilo-dessi/SKG-pipeline-cskg-plus. All the information about the graph can also be found on a dedicated website https://w3id.org/cskg.
